# War on Disease

**Published:** 1920-04-24

**Authors:** 


THE PUBLIC WELL-BEING—(*continued*)

### WAR ON DISEASE.

### International Conference in London.

The Commission which had met informally on the invitation of the British Government in July 1919 to discuss certain questions of international concern in connection with health, was invited by the Council of the League of Nations to constitute a conference by adding to its members a small number of international health experts, with an official of the League as secretary. This Conference was to prepare for submission to the Council proposals concerning the constitution of a permanent body to whom the Council might refer for advice, and, if necessary, for action on all health questions within the scope of the League. The Conference, constituted in accordance with the wish expressed by the Council, had its first session in London on April 13, and concluded its business last Saturday. Viscount Astor was the chairman, and the Conference consisted of delegates from France, Great Britain, Italy, Japan, the United States, the League of Red Cross Societies, and the Office International d'Hygiène Publique.

The scheme agreed upon for submission to the Council of the League of Nations is broadly on the lines of the International Labour Office, which means that there will be a permanent international health organisation as part of the League, with its secretariat, its executive committee, and a general committee acting in some degree like an international health parliament. The executive committee will meet at least four times a year, and the general committee at least once a year. The main functions of this permanent organisation will be to advise the League in matters affecting health; to bring administrative health authorities in different countries into close relationship with each other; to organise means of rapid interchange of information on matters whereon immediate precautions against disease may be required, as, for example, in epidemics; and to simplify methods for acting rapidly on such information where it affects more than one country; to provide a ready organisation for securing or revising necessary international agreements; and, broadly, to confer and co-operate with the League of Red Cross Societies and other health organisations. It is proposed also that it should advise, when requested, authorised voluntary organisations in health matters of international concern. In regard to measures for the protection of the worker against disease, sickness and injury arising out of his employment which fall within the province of the International Labour Organisation, the International Health Organisation will co-operate with, and assist the Labour Organisation. The International Labour Organisation on its part will act in consultation with the International Health Organisation in regard to all health matters.

The permanent organisation will incorporate the Bureau and Office International d'Hygiène Publique, which up to the present has been the official international health organisation. The Bureau, which will be the Secretariat of the Organisation, will include a medical secretary, and its headquarters will be at the seat of the League.

Lord Astor, in an interview, emphasised the importance of the League's initiative, and the hope of far-reaching results not only in preventing and combating the spread of disease, but also in improving the general welfare of all countries and in establishing better understanding. Such work as the international care of health and of conditions of labour would do much in assisting to make the League an influential and living organism.

The representatives of Great Britain at the Conference were Sir George Newman, Dr. G. S. Buchanan, and Dr. Steegmann (Technical Adviser). Surgeon-General Rupert Blue was the United States' delegate, and the League of Red Cross Societies was represented by General Sir David Henderson and Colonel R. P. Strong, U.S.A. (Technical advisor). A member of the Secretariat of the League of Nations, Dame Rachel Crowdy, R.R.C., acted as secretary.

